# Allogeneic Umbilical Cord-Derived Mesenchymal Stem Cells as a Potential Source for Cartilage and Bone Regeneration: An *In Vitro* Study

**DOI:** 10.1155/2017/1732094

**Published:** 2017-11-16

**Authors:** A. Marmotti, S. Mattia, F. Castoldi, A. Barbero, L. Mangiavini, D. E. Bonasia, M. Bruzzone, F. Dettoni, R. Scurati, G. M. Peretti

**Affiliations:** ^1^Department of Orthopaedics and Traumatology, University of Turin, Torino, Italy; ^2^University of Turin Molecular Biotechnology Center, Torino, Italy; ^3^Department of Biomedicine, University Hospital of Basel, University of Basel, Basel, Switzerland; ^4^IRCCS Istituto Ortopedico Galeazzi, Milano, Italy; ^5^Department of Biomedical Sciences for Health, University of Milan, Milano, Italy

## Abstract

Umbilical cord (UC) may represent an attractive cell source for allogeneic mesenchymal stem cell (MSC) therapy. The aim of this *in vitro* study is to investigate the chondrogenic and osteogenic potential of UC-MSCs grown onto tridimensional scaffolds, to identify a possible clinical relevance for an allogeneic use in cartilage and bone reconstructive surgery. Chondrogenic differentiation on scaffolds was confirmed at 4 weeks by the expression of sox-9 and type II collagen; low oxygen tension improved the expression of these chondrogenic markers. A similar trend was observed in pellet culture in terms of matrix (proteoglycan) production. Osteogenic differentiation on bone-graft-substitute was also confirmed after 30 days of culture by the expression of osteocalcin and RunX-2. Cells grown in the hypertrophic medium showed at 5 weeks safranin o-positive stain and an increased CbFa1 expression, confirming the ability of these cells to undergo hypertrophy. These results suggest that the UC-MSCs isolated from minced umbilical cords may represent a valuable allogeneic cell population, which might have a potential for orthopaedic tissue engineering such as the on-demand cell delivery using chondrogenic, osteogenic, and endochondral scaffold. This study may have a clinical relevance as a future hypothetical option for allogeneic single-stage cartilage repair and bone regeneration.

## 1. Introduction

Cartilage and bone lesions represent a common problem in the orthopaedic practice, and tissue engineering is constantly proposing innovative approaches to improve their repair. Current treatments for cartilage defects are bone marrow stimulation (microfractures), autologous osteochondral transplantation, and autologous chondrocyte implantation. However, these options have specific limitations and disadvantages: the poor quality of the repair tissue, the donor-site morbidity, and the limited availability of tissue [1]. For bone repair, the available bone substitutes are acellular and do not possess any osteogenic potential, representing simple “gap-filling scaffold” to be populated by resident cells.

To overcome these issues, the use of autologous mesenchymal stem cells (MSCs) has gained popularity due to the ability of these cells to differentiate toward chondrogenic or osteogenic pathways. Generally, MSCs are derived from bone marrow aspirations or from lipoaspirates, which contain an undifferentiated population of precursors, both CD34^+^ and CD34^−^ along with a great number of blood mononuclear cells: These cell concentrates are currently used for one-stage treatment of cartilage or bone defects [2–7]. The main disadvantage of this approach is the limited number of MSCs in the final product [8]. Thus, the use of selected and precultured MSCs is under investigation [9]. In this perspective, allogeneic cells would eliminate the morbidity of harvesting procedures and the costs linked to these procedures. Indeed, a cell factory may host a great number of selected allogeneic stem cell lines from different donors, readily available for clinical use.

Besides the well-known sources as the bone marrow and the fat, new allogeneic cell sources are emerging, such as the umbilical cord stroma (UC) [8, 10–12]. The application of cells derived from UC structure has some nonnegligible advantages compared to other sources; these cells are indeed isolated from a formerly discarded material that has a virtual unlimited availability [12]. Moreover, UC contains two umbilical arteries and one umbilical vein and a mucous proteoglycan-rich connective tissue, named Wharton's jelly, covered by amniotic epithelium: Stem cells may be isolated from each of these structures with a promising efficiency [10, 13]. These cells have unique properties compared to other stem cell types as they lie between embryonic stem cells (ESCs) and adult mesenchymal stem cells (MSCs) on the development map, they share stemness markers with ESCs and MSCs, they do not induce tumorigenesis, and they are hypoimmunogenic [14]. When taken together, the different UC-MSC subtypes constitute a “mixed” heterogeneous MSC population, which is able to differentiate toward the osteogenic, adipogenic, or chondrogenic lineage [15].

Thus, UC-MSCs may represent an appealing cell source with a potential for clinical allogeneic use to treat chondral, osteochondral lesions, and bone defects, being a possible candidate for a “universal off-the-shelf” stem cell product in the field of orthopaedic tissue engineering [13].

The goal of this *in vitro* study was to evaluate the capability of allogeneic UC-MSCs to differentiate toward chondrogenic or osteogenic pathway in a tridimensional environment and to test the possibility to address these cells toward a hypertrophic stage, as a first step to recapitulate the endochondral ossification.

## 2. Materials and Methods

Approvals were obtained both from the Ethical Committee of MBC (Molecular Biotechnology Center), University of Turin, and from the Ethical Committee of Mauriziano Hospital, Turin (Italy); protocol number is CS792 approved on January 11, 2016.

### 2.1. UC Collection and Processing

After obtaining patient's informed consent, 15 fresh UC samples were retrieved during caesarean deliveries from the Department of Obstetrics and Gynecology of Mauriziano Hospital (Turin, Italy). The UC samples were collected in a phosphate-buffered saline (PBS) transfer medium containing 200 mg/100 ml ciprofloxacin, 500 IU heparin, and they were immediately processed. After transferring samples under a sterile laminar flow cell culture hood, the cord length and weight were estimated and the UC was washed in PBS to remove traces of contaminant red blood cells. The UC was first cut into 3 cm long segments, which were subsequently cut longitudinally and split open to expose the inner surface.

The UC segments were then manually minced into small cuboidal fragments (4–7 mm length). The fragments were seeded in 60 cm^2^ Petri dishes with the same expansion medium in which they have been minced. This mesenchymal stem cell expansion medium contained Dulbecco's Modified Eagle Medium/F-12 (DMEM) enriched with 5% human platelet lysate obtained from healthy donors, 10% fetal bovine serum (FBS), 1X penicillin/streptomycin, 1X sodium pyruvate, 1X nonessential amino acids, 500 IU heparin (Pharmatex). The small UC fragments were distributed into 6–7 different 60 cm^2^ Petri dishes (approximately 40–45 fragments/Petri dish) and incubated in the MSC expansion medium at 37°C in a humidified atmosphere with 5% CO_2_ (day 0). Fragments of UC were left undisturbed in culture and monitored for up to 2 weeks to allow identification of MSC in the dishes. Cell isolation was successful for 11 samples out of 15.

### 2.2. UC-MSC Culture

After 2 weeks (day 14), the UC debris were removed and adherent cells were expanded for 2 additional weeks; cell expansion was reached for 11 samples out of 15. Forty percent of the medium was changed every 3–4 days. After 2 weeks, the adherent cells (P0) were trypsinized, centrifuged at 1200 rpm for 10 min, resuspended in the MSC expansion medium, and replated for one consecutive expansion step at a density of 100–200 cells/cm^2^, until full confluence was reached (P1). Cell confluence at P1 was reached after approximately 14 days (day 42). At the end of P1 passage (day 42), living cells were counted by trypan blue dye exclusion.

### 2.3. Telomere Length Analysis

Telomere length was evaluated on UC-MSCs at P1 from 4 UCs, and results were compared to telomere length of the same cell line at sequential passages from P2 to P5, following a previously reported method [15].

### 2.4. UC-MSC Immunophenotypic Characterization

Immunophenotyping of the expanded UC-MSCs was done by flow cytometry analysis at P1. 1,5 × 10^6^ UC-MSCs were used for flow cytometry. The following antibodies were used: CD90-peridinin chlorophyll protein- (PerCP-) cyanine dye Cy5.5 (Biolegend, San Diego, CA), CD105-fluorescein isothiocyanate (FITC) (Biolegend, San Diego, CA), CD73-allophycocyanin (APC) (BD Biosciences, San Jose, CA), CD34-phycoerythrin (PE) (BD Biosciences, San Jose, CA), HLA-DR-FITC (BD Biosciences, San Jose, CA), HLA-PerCP (BD Biosciences, San Jose, CA), HLA-ABC-PE, CD29-APC (BD Biosciences, San Jose, CA), CD44-Alexa Fluor (Cell Signaling Technology, Danvers, MA), PE-conjugated antimouse immunoglobulin G (IgG) (Southern Biotechnology Associates, Birmingham, Alabama, USA), isotype-matched IgG-FITC (Biolegend, San Diego, CA), IgG-PE (Biolegend, San Diego, CA), and IgG-PE-Cy5 (Biolegend, San Diego, CA) control antibodies. Analysis was performed on a FACScan (Becton Dickinson (BD), Buccinasco, Italy) for at least 10.000 events and using CellQuest software (BD, Buccinasco, Italy).

### 2.5. Section 1: UC-MSC Differentiation on Chondrogenic Scaffold

UC-MSCs at P1 were assessed for chondrogenic differentiation on scaffolds. UC-MSCs were loaded onto two different scaffolds: Hyaff-11 (FIDIA Advanced Biopolymers, Italy) or Chondro-gide (Geistlich Biomaterials, Italy S.r.l.) membranes. Hyaff-11 is a nonwoven esterified HA derivative membrane. Chondro-gide is a double-layer matrix of pig collagen type I and type III, with a smooth compact side and a porous side, where cells are seeded. After the first passage of cell culture, 2 × 10^6^ UC-MSCs were resuspended in 50 *μ*l of the chondrogenic differentiation medium and seeded onto a scaffold surface of 1 cm^2^ (either Hyaff-11 or Chondro-gide). The scaffolds were left at 37°C in a humidified atmosphere with 5% CO_2_ for 3–4 hours to allow UC-MSC adhesion on the scaffolds. Then, two drops of commercial fibrin glue (Tissucol-Tisseel, Baxter) were added as a surface sealing, and the final constructs were incubated at 37°C, in a humidified atmosphere for 1 month in the presence of the chondrogenic differentiation medium (EUROMED Chondrogenic Differentiation Kit, EuroClone, Pavia, Italy). Cultures were performed both in normoxic conditions (21% O_2_) or at low oxygen tension (8% O_2_).

After 1 month, constructs were fixed in formalin, included in paraffin, and sectioned. Sections were stained for haematoxylin/eosin and examined under light microscopy. For cell counting, three different areas in two different sections per construct were examined under light microscopy at 20x magnification by two independent observers. Cell number for each single area was defined by the arithmetical mean of the cell counts from both observers. Mean numbers of migrating cells from every area were statistically compared and graphed with GraphPad Prism®. Safranin o staining was also performed on sections.

Expression of chondrocyte markers, sox-9 (AB5535, Merck Millipore, Milano, Italy), and collagen type II (clone 6B3, MAB8887, Merck Millipore, Milano, Italy) was assessed using immunofluorescence techniques. The primary monoclonal antibodies were diluted in PBS-BSA1% and incubated with the sections for 2 h at room temperature. The secondary dye light 488 antibody (KPL, Kirkegaard & Perry Laboratories, Maryland, USA), diluted 1 : 100, was incubated for 1 h at room temperature. The stained sections were visualized with an Apotome fluorescence microscope. We collected digital images using a ×20 dry lens within 0–5 days after labelling.

The same culture conditions were used for UC-MSC pellet culture. Proteoglycan (PG) : DNA ratio was calculated as the best approximation of ECM production per cell following a previously reported method [16].

### 2.6. Section 2: UC-MSC Differentiation in Osteogenic Scaffold

For osteogenic differentiation on scaffold, we used the Orthoss® bone graft (Geistlich Biomaterials, Italy S.r.l.), a bovine-derived natural commercial bone substitute. Its inorganic bone matrix has a macro- and microporous structure similar to human cancellous bone. Scaffolds were cut into cubes of approximately 1 cm^3^. The cubes were then coated with fibronectin by soaking in a solution containing 50 mg/ml fibronectin for 4 h at room temperature. The cubes were air dried overnight in a sterile bio safety cabinet. The cubes were seeded with UC-MSCs resuspended in fibrin glue (6 × 10^6^ cells/scaffold). Then the osteogenic differentiation medium was added (EUROMED Osteogenic Differentiation Kit, EuroClone, Pavia, Italy), and constructs were incubated at 37°C in a humidified atmosphere with 5% CO_2_ for 10, 20, and 30 days.

At the end of culture, constructs were initially decalcified and then fixed in formalin, included in paraffin, and sectioned. Sections were stained for haematoxylin/eosin and Alizarin red and examined under light microscopy. Expression of osteocalcin ([Tyr28-, Phe42-, and Phe46-] bone Gla protein, Phoenix Pharmaceuticals Inc., Burlingame, CA, USA) and core-binding factor subunit alpha-1/runt-related transcription factor 2 Cbfa1/RunX-2 (Ab 114,133, Abcam, Cambridge, MA, USA) markers was assessed using immunofluorescence techniques described above. The stained sections were visualized with an Apotome fluorescence microscope. We collected digital images using a × 20 dry lens within 0–5 days after labelling.

### 2.7. Section 3: UC-MSC Hypertrophic Differentiation

To verify the UC-MSC potential for endochondral differentiation, cells at P2 were seeded onto Orthoss 3 g granules of approximately 4 mm^3^ of volume (concentration: 25 × 10^4^ cells/granule) and incubated at 37°C in a humidified atmosphere with 5% CO_2_, in three different conditions:

- Hypertrophic culture (3 weeks + 2 weeks)
  1. Chondrogenic medium (Chondrogenic Differentiation Kit, EuroClone), for 3 weeks
  2. Hypertrophic medium (DMEM, 10 mM Hepes Buffer, 1 mM Na pyruvate, 1% penicillin/streptomycin/glutamine, 1% ITS-A, 4.7 *μ*g/ml linoleic acid, 1.25 mg/ml human serum albumin, 0.1 mM ascorbic acid 2-phosphate, 10^−8^ M dexamethasone, 10 mM *β*-glycerophosphate, 0.05 *μ*M L-thyroxin) for the following 2 weeks
- This peculiar condition was compared with two other culture conditions:
- Osteogenic culture (*α*MEM, 10% fetal calf serum, 0.1 mM ascorbic acid 2-phosphate, 10^−8^ M dexamethasone, 10 mM *β*-glycerophosphate) for 3 and 5 weeks
- Basal (control) culture: DMEM +10% fetal calf serum, 50 U/ml penicillin/streptomycin for 3 and 5 weeks

At the end of culture (3 and 5 weeks), H&E and safranin o staining were performed, following the manufacturer's protocols (Bio-Optica Milano SpA, Milan, Italy). Furthermore, real-time PCR analysis was completed to analyze gene expression of the major chondrogenic and osteogenic markers (sox-9, Cbfa1).

#### 2.7.1. RT-PCR Analyses

Total RNA was extracted from constructs using TRIzol® (Life Technologies), and cDNA was generated as previously described [17]. The PCR master mix was based on AmpliTaq Gold DNA polymerase (Perkin Elmer/Applied Biosystems). TaqMan® gene expression or on-demand assays (Life Technologies) were used on a ABI 7900 fat real-time PCR system (Life Technologies) for 40 cycles to measure gene expression of sox-9 (Hs00165814_m1) and Cbfa1 (Hs00231692_m1) using GAPDH (Hs99999905_m1) as the housekeeping gene.

### 2.8. Evaluation of Fluorescence Intensity

The difference of fluorescence intensity between the constructs was evaluated using ImageJ program. This software generated numerical semiquantitative evaluations corresponding to the mean fluorescence intensity of each image examined. Ten cellular fields were randomly chosen among the different areas of migrated chondrocytes in each slide. Briefly, a point tool enables the marking of locations on an image. With each “click,” the coordinates of the mark (*xx*, *yy*) and brightness values (0–255) are recorded in a data window. ImageJ brightness units are in a scale where 0 = pure black and 255 = pure white. Brightness values for each image were calculated as the arithmetical mean of all values in all fields recorded for that image. For each construct, mean fluorescence intensity of each marker was calculated and plotted as a graph. The difference in intensity allowed for evaluating the change in marker expression between the different culture conditions.

### 2.9. Statistical Analysis

All data in text and figures are provided as medians. Statistical analysis was carried out with the statistical software package GraphPad Prism 5.0. The results are shown as box plots, where the transverse line represents the median value, and the width of the box is given by the minimum and the maximum value of the data. If only two conditions are compared, we used Mann–Whitney test and we did not assume Gaussian distribution; if more than two conditions are compared, we used one-way ANOVA and Bonferroni adjustment.

## 3. Results

### 3.1. UC-MSC Morphologic and Immunophenotypic Characterization

**Figure 1:**
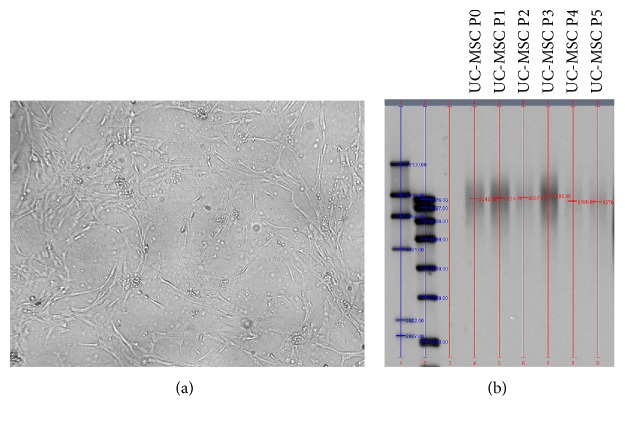
UC-MSC morphological characterization. (a) UC-MSCs at P1. Cells show a fibroblast-like morphology. Magnification 10x. (b) UC-MSC telomere length analysis at different passages (between P1 and P5); no difference is detected; *n* of samples = 4.

In primary cultures, typical spindle-shaped adherent cells were observed migrating from the UC tissue fragments and initiating colony formation approximately at day 14 after seeding. After removing the UC fragments at day 14 postseeding, cells took approximately 10 days to gain 60% confluence (Figure 1(a)), while full confluence was observed approximately at day 28 postseeding. The UC-MSC clones (defined as passage P0) were thus collected at day 28 postseeding and replated for further expansion (defined as passage P1). Confluence at P1 was observed approximately after 14 days of culture (day 42 postseeding).

At day 42 (confluence at P1 passage), we obtained a mean of 23.05 × 10^6^ (SD 1.48) cells from each umbilical cord. From the initial seeding (day 0), we obtained at the end of the P1 (day 42) 0.80 × 10^6^ (SD 0.28) cells/g of UC seeded (mean weight of the UC 30.65 g − mean length 40.9 cm) (Supplemental Figure 1 available online at https://doi.org/10.1155/2017/1732094). UC cells' phenotype was analyzed by flow cytometry. The majority of collected UC cells showed a positive expression of the main MSC markers CD73, CD90, and CD105, as well as of CD44 and CD29. Furthermore, they stained negative for the typical hematopoietic marker CD34 (Supplemental Figure 2). The data also demonstrated the presence of HLA-ABC proteins and the absence of HLA-DR. Additionally, we visualized a notable presence (40%) of negative double cells for both HLA-ABC and HLA-DR proteins.

Telomere length analysis performed on UC-MSCs at different culture passages (from P1 to P5) did not show any significant difference (Figure 1(b)).

### 3.2. Section 1: Chondrogenic Differentiation in Scaffold

**Figure 2:**
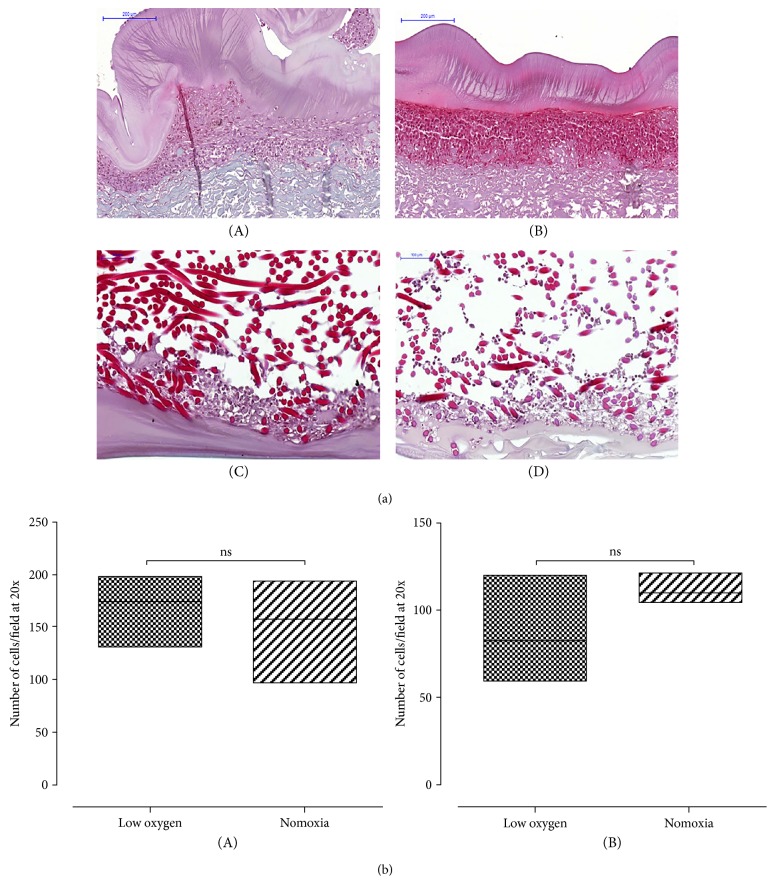
Chondrogenic differentiation of UC-MSCs seeded on a scaffold. (a) A representative image of safranin o staining of UC-MSCs seeded onto Chondro-gide (A and B) or Hyaff (C and D) membranes and cultured in normoxic (A and C) or low oxygen tension conditions (B and D). UC-MSCs differentiate inside the three-dimensional structure of the HYAFF-11, while they are confined to the surface of the Chondro-gide scaffold. A stronger safranin o staining is observed in the low oxygen tension constructs than in normoxic culture. Magnification 20x. Scale bar: 200 *μ*m in (A and B); 100 *μ*m in (C and D). (b) No significant difference in cell number per field is observed comparing normoxic and in low oxygen tension conditions in Chondro-gide (A) (median value and standard deviation: 174 ± 32.07; 157.5 ± 40.32 for low oxygen tension and normoxia, resp.) and Hyaff (B) (median value and standard deviation: 82.50 ± 24.96; 110 ± 6952 for low oxygen tension and normoxia, resp.) scaffolds. *p* value > 0.05; *n* of samples = 3.

During chondrogenic differentiation onto Chondro-gide and Hyaff-11 scaffolds, cells showed chondrogenic commitment both in normoxic conditions and in low oxygen tension, albeit UC-MSCs cultured at lower oxygen tension showed more positive safranin o staining, consistent with increased sulfated glycosaminoglycan (s-GAG) production (Figure 2(a), C and D). Furthermore, histological analysis showed that MSCs migrated inside the tridimensional structure of the Hyaff-11 scaffold, while they remained almost complete at the porous surface on the Chondro-gide membrane (Figure 2(a), A and B), probably due to the different composition of the scaffolds. No significant difference in the number of cells per field was observed between normoxic cultures and low oxygen tension conditions for each specific scaffold (Figure 2(b)).

**Figure 3:**
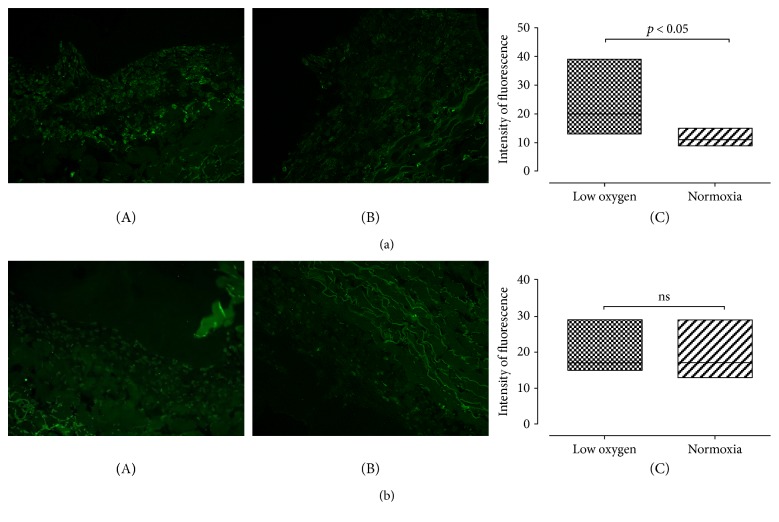
Chondrogenic differentiation of UC-MSCs seeded on Chondro-gide. (a) A representative image of immunofluorescence staining for sox-9 in Chondro-gide scaffold at low oxygen tension (A) and normoxic (B) conditions. Quantification of the signal is shown in (C): Fluorescence intensity is significantly higher in constructs grown at low oxygen tension (median value and standard deviation: 20 ± 6122; 11 ± 2102 for low oxygen tension and normoxia, resp.). *p* value < 0.05. Magnification 20x; *n* of samples = 3. (b) A representative image of immunofluorescence staining for collagen type II in Chondro-gide scaffold at low oxygen tension (A) and normoxic (B) conditions. Signal quantification is shown in (C); no significant difference in fluorescence intensity is observed (median value and standard deviation: 17 ± 3279; 17 ± 4025 for low oxygen tension and normoxia, resp.). *p* value = 0.6926. Magnification 20x; *n* of samples = 3.

In Chondro-gide scaffolds, positive immunostaining for sox-9 was present both in normoxic culture and at low oxygen tension conditions (Figure 3(a), A and B). A significantly higher fluorescence intensity was observed in constructs cultured art low oxygen tension (*p* value  < 0.05) (Figure 3(a), C). Collagen type II expression in Chondro-gide scaffold was present both in low oxygen tension and under normoxic conditions (Figure 3(b), A and B); however, fluorescence intensity was not significantly different (*p* value = 0.6926) (Figure 3(b), C). Negative controls are shown in Supplemental Figure 3.

**Figure 4:**
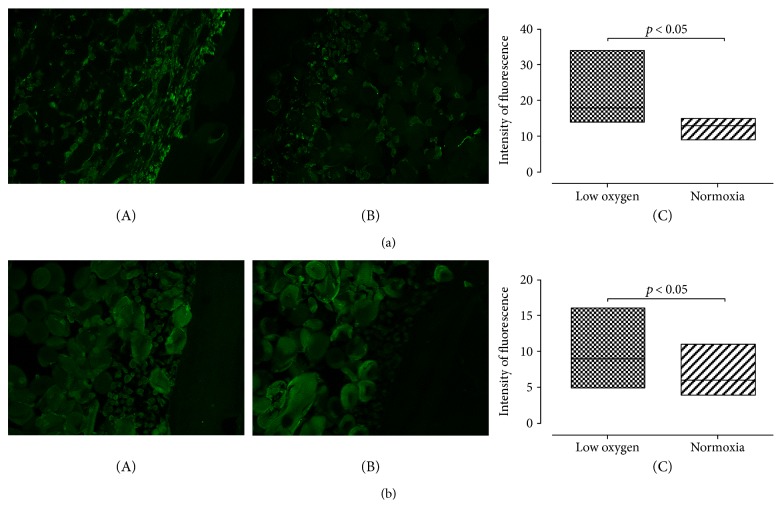
Chondrogenic differentiation of UC-MSCs seeded on Hyaff. (a) A representative image of immunofluorescence staining for sox-9 in Hyaff scaffold at low oxygen tension (A) and normoxic (B) conditions. Quantification of the signal is shown in (C): Fluorescence intensity is significantly higher in constructs grown at low oxygen tension (median value and standard deviation: 18 ± 5194; 13 ± 1917 for low oxygen tension and normoxia, resp.). *p* value < 0.05. Magnification 20x; *n* of samples = 3. (b) A representative image of immunofluorescence staining for collagen type II in Hyaff scaffold at low oxygen tension (A) and normoxic (B) conditions. Signal quantification is shown in (C): Fluorescence intensity is significantly higher in constructs grown at low oxygen tension (median value and standard deviation: 9 ± 2601; 6 ± 1594 for low oxygen tension and normoxia, resp.). *p* value < 0.05. Magnification 20x; *n* of samples = 3.

In Hyaff-11 scaffolds, we noticed a similar trend: Positive immunostaining for sox-9 was noticed both at low oxygen tension and in normoxic cell cultures (Figure 4(a), A and B). The difference in fluorescence intensity resulted statistically significant (*p* value < 0.05) (Figure 3(a), C). Collagen type II expression in Hyaff-11 scaffolds was significantly greater at low oxygen tension (*p* value < 0.05) (Figure 4(b), C). Negative controls are shown in Supplemental Figure 4.

**Figure 5:**
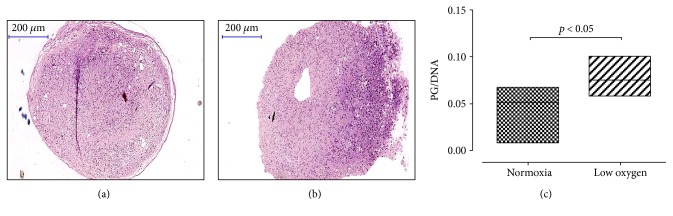
UC-MSC chondrogenic differentiation in pellet culture. A representative image of safranin o staining of UC-MSCs in pellet culture (a and b). Stronger safranin o staining is observed in UC-MSC pellets cultured at low oxygen tension (a) when compared to normoxic conditions (b). Higher PG/DNA ratio in pellets cultured at low oxygen tension is shown in (c) (median value and standard deviation: 0.05166 ± 0.02240; 0.07468 ± 0.01687 for and normoxia and low oxygen tension, resp.). *p* value < 0.05. Magnification 20x. Scale bar: 200 *μ*m. *n* of samples = 3.

A similar trend was observed in UC-MSC pellet cultures when exposed to low oxygen tension during culture period. We observed a stronger safranin o staining (Figures 5(a) and 5(b)) and higher PG/DNA ratio (Figure 5(c)) in pellet culture grown at low oxygen tension compared to those exposed to normoxic conditions (*p* value < 0.05).

### 3.3. Section 2: Osteogenic Differentiation in Bone Substitutes

**Figure 6:**
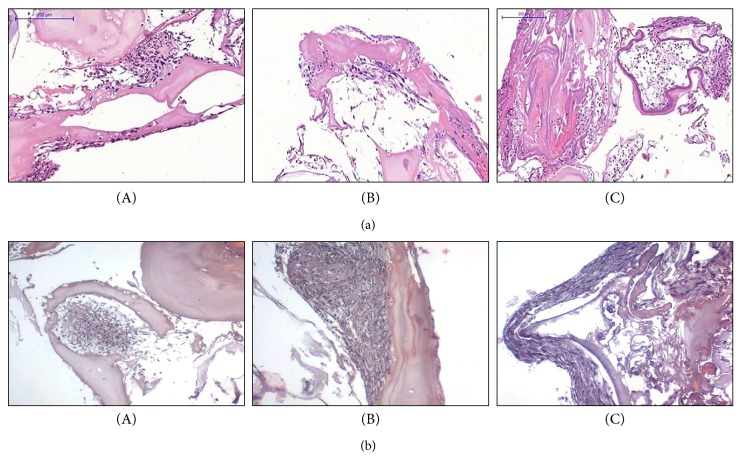
UC-MSC osteogenic differentiation on the Orthoss scaffold. (a) A representative image of H&E staining of UC-MSCs loaded onto Orthoss scaffold at 10 (A), 20 (B), and 30 (C) days of in vitro culture. Magnification 20x. Scale bar: 200 *μ*m. (b) A representative image of Alizarin red staining of UC-MSCs loaded onto Orthoss scaffold at 10 (A), 20 (B), and 30 (C) days of in vitro culture. Magnification 20x. Scale bar: 100 *μ*m.

**Figure 7:**
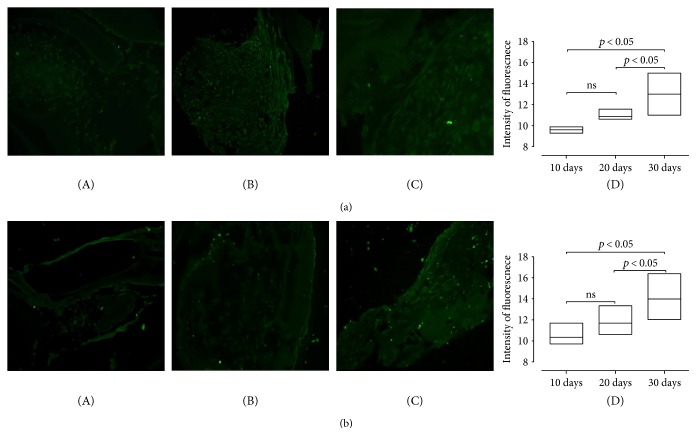
Expression of osteogenic markers in UC-MSCs seeded onto Orthoss cubes. (a) A representative image of immunofluorescence staining for osteocalcin in Orthoss at 10 (A), 20 (B), and 30 (C) days of *in vitro* culture. Quantification of the signal is shown in (D): Fluorescence intensity is significantly higher in samples cultured for 30 days (median value and standard deviation: 9604 ± 0.2584; 10.85 ± 0.3990; 13 ± 1030 in samples cultured for 10, 20, or 30 days, resp.). *p* value < 0.05. Magnification 20x; *n* of samples = 5. (b) A representative image of immunofluorescence staining for RunX-2 in Orthoss at 10 (A), 20 (B), and 30 (C) days of *in vitro* culture. Quantification of the signal is shown in (D): Fluorescence intensity significantly increases in the longer experimental time points (median value and standard deviation: 7 ± 1340; 11 ± 1712; 18 ± 3090 in samples cultured for 10, 20, or 30 days, resp.). *p* value < 0.05. Magnification 20x; *n* of samples = 5.

Scaffolds showed a considerable increasing cellularity in constructs at different time points (10, 20, and 30 days) (Figure 6(a)). Alizarin red stain showed calcium deposits gradually increasing from 10, 20, to 30 days (Figure 6(b)). Semiquantitative analysis of osteocalcin immunostaining (Figure 7(a), A–C) showed a significant increased intensity of osteocalcin expression between 20 days and 30 days; for the other time points, there was an increased expression, though it did not reach a significant difference (Figure 7(a), D; *p* value < 0.05). Similar results were obtained for the expression of the transcriptional factor RunX-2, (Figure 7(b), A–C) which is a key transcription factor associated with osteoblast differentiation (Figure 7(b), D) (*p* value < 0.05). Negative controls are shown in Supplemental Figure 5.

### 3.4. Section 3: Endochondral Differentiation in Bone Substitute Granules

**Figure 8:**
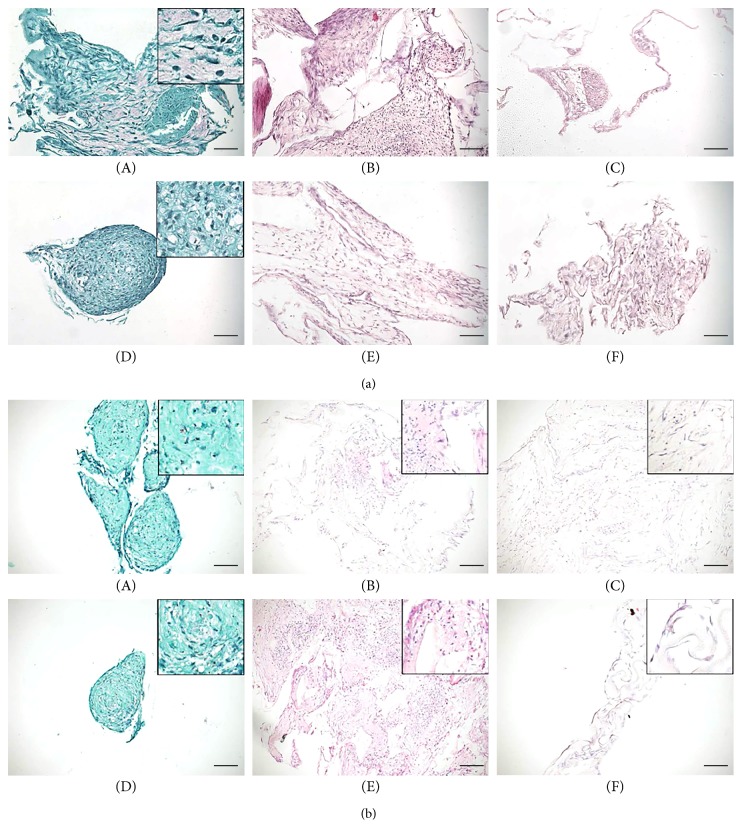
Hypertrophic differentiation of UC-MSCs seeded onto Orthoss granules. (a) A representative image of safranin o and H&E staining of UC-MSCs seeded onto Orthoss granules and cultured for 3 weeks in the hypertrophic medium (Safranin O stain, A and D), osteogenic medium (H&E stain, B and E) or basal medium (H&E stain, C and F), respectively. Scale bar: 100 *μ*m; *n* of samples = 5. (b) A representative image of safranin o and H&E staining of UC-MSCs seeded onto Orthoss granules and cultured for 5 weeks in the hypertrophic medium (Safranin O stain, A and D), osteogenic medium (H&E stain, B and E), or basal medium (H&E stain, C and F), respectively. Scale bar: 100 *μ*m; *n* of samples = 5.

At 3 weeks of culture, deposition of a cartilaginous matrix was observed in the samples cultured with the chondrogenic induction medium, while there was no evidence of osteogenic differentiation in specific differentiation medium (Figure 8(a)). At 5 weeks of culture, we observed a stronger safranin o staining in samples cultured in the chondrogenic hypertrophic medium, as well as the production of bone matrix in samples cultured in the osteogenic differentiation medium (Figure 8(b)).

**Figure 9:**
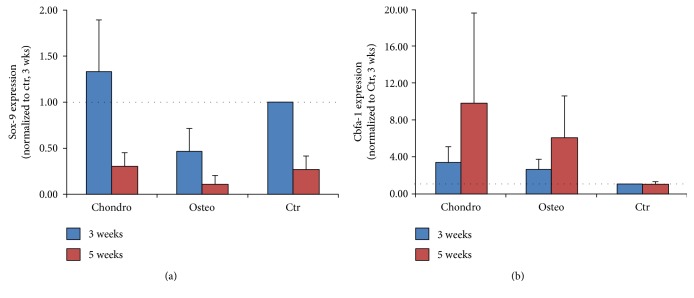
Expression of osteogenic markers in UC-MSCs undergoing hypertrophy. Quantification of sox-9 (a) and Cfba-1 (b) gene expression by RT-PCR analysis at 3 or 5 weeks of culture in the three experimental groups. GAPDH used as gene of reference; *n* of samples = 5.

Real-time PCR analysis showed a mild upregulation of sox-9 expression in samples cultured for 3 weeks in the chondrogenic medium. Also, the osteogenic marker Cfba-1 was upregulated in samples cultured both in chondrogenic and in the osteogenic medium. After 5 weeks of culture, sox-9 expression was downregulated in samples cultured with the chondrogenic hypertrophic medium, whereas Cfba-1 was upregulated. The osteogenic medium induced a strong Cfba-1 upregulation (Figure 9).

## 4. Discussion

The main finding of this study is that UC-MSCs collected in a straightforward and simple procedure from minced umbilical cord fragments may be committed toward chondrogenic and osteogenic lineages, when cultured in scaffolds, and they may also be addressed toward hypertrophy when cultured in bone substitute in the presence of the chondrogenic and hypertrophic chondrogenic medium. Thus, UC-MCs may represent an allogeneic cell population with a promising value for on-demand cell delivery in single-stage cartilage repair and bone regeneration.

Nowadays, the use of allogeneic cells for cartilage and bone repair is an ongoing frontier due to the increasing need of cells for better specific tissue repair.

Indeed, the simple use of bone marrow stimulating techniques, such as microfracture, has shown some limitations linked to the restricted durability of the repair and the lesser quality of the tissue obtained, compared to that achieved by more complex reconstructive techniques as autologous chondrocyte implantation [18–20] or scaffold-driven repair enriched by autologous bone marrow MSCs [21]. Even the use of bone substitutes has shown lesser results when compared with techniques combining bone substitute with biological stimuli as autologous bone marrow MSCs or autologous stem cell mobilization through growth factors as G-CSF (granulocyte colony stimulating factor) [22, 23]. However, the use of an autologous cell source has several disadvantages mainly due to the morbidity of the harvesting procedure, the individual variability in the cell number, and the limited number of cells available by each harvesting procedure. Moreover, the use of selected precursor cells (i.e., precultured autologous stem cells, chondrocytes) is necessarily associated with multiple procedures to obtain the primary autologous source, to culture the cells and to reimplant the cells at the lesion site. An ongoing solution proposed in literature is the “one-step procedures” in which autologous unselected sources of cells, as bone marrow concentrate [24], cartilage fragments [1, 25], or stromal vascular fraction from lipoaspirates [5] are added at the lesion site (cartilage or bone defect) obtaining interesting results, even if in these instances, a noncommitted cell population is used to enhance the repair.

In line with the concept of “one-step procedure,” the evolving technologies in cell storage and cell culture allow for hypothesizing the use of allogeneic cells as an attractive choice for cartilage and bone repair, due to the greater bioavailability of allogeneic sources compared to the autologous ones. Thus, the concept of allogeneic stem cell therapy is becoming an ongoing reality. Indeed, in several clinical fields, such as neurology, gastroenterology, or hematology, these principles are used in experimental studies to treat different diseases like cerebral palsy [26], autoimmune encephalomyelitis [27], perianal fistulas in Crohn's disease [28], liver failure [29], and aplastic anemia [30]. The main advantages in the use of allogeneic cells in orthopedics are a greater “on-demand” availability of cell precursors, the absence of harvesting morbidity, the possibility to obtain a selected cell population, and even the possibility to use cells from younger donors for lesions in older recipient, further optimizing the quality of the repair. The latest aspect has recently found a preclinical application in the study of Bonasia et al. [1], in which allogeneic juvenile cartilage fragments have been used to improve the quality of cartilage repair in a rabbit model obtaining positive results. This work suggests the possibility to use juvenile fresh allogeneic tissue grafts as a source for the repair. This concept is certainly an option for bone and cartilage reconstruction, but it implies several drawbacks as the prompt bioavailability of the specific tissues and the costs of tissue preservation, posing some concerns about a widespread application of these principles.

A different solution is to use allogeneic candidates from different anatomical sites as bone marrow and adipose tissue. In the *in vivo* study by de Windt et al. [31], cartilage defects are treated by combining allogeneic bone marrow MSCs with autologous chondrons obtained by digesting minced cartilage fragments. Result of this phase I study seems promising, though in that case, the length of the entire procedure (i.e., obtaining autologous chondrons + combination of the 2 sources of repair + surgical implantation) may represent a drawback, if compared to more “straightforward” procedures in which a cellularized scaffold is directly implanted at the lesion site. In preclinical rabbit and minipig models, allogeneic bone marrow-derived MSC implantation has been proposed for the treatment of knee osteochondral defects with promising results [32, 33]. Bone regeneration has been obtained in a study by Kang et al. [34] loading allogeneic bone marrow MSCs onto allogeneic cancellous bone granules in a rabbit radial defect model, with a quality of repair comparable to that with autologous BM-MSCs. In several preclinical studies, allogeneic adipose-derived MSCs (ASCs) have also improved bone and cartilage repair. In the study conducted by Wen et al. [35], ASCs have been combined with demineralized bone matrix (DBM) have been applied in ulnar bone defects in rats with promising results. Even the intra-articular injection of allogeneic, ASCs have led to satisfying outcomes when combined with hyaluronic acid in preclinical models of dog arthropathy [36, 37] and sheep osteoarthritis [38].

The umbilical cord-derived MSCs (UC-MSCs) may represent a novel interesting alternative in this field [39]. The umbilical cord is considered a discarded material, and its use implies fewer ethical and legal issues than the other embryonic structures (i.e., embryonic stem cells). Furthermore, it has a virtually “unlimited availability,” as recently defined by Kalaszczynska and Ferdyn [8]. Moreover, the low costs and the absence of morbidity related to the collecting procedure are a “nonnegligible” advantage compared to the other common allogeneic cell sources, such as bone marrow and adipose tissue.

In literature, UC-MSCs have shown a favorable *in vitro* potential as they can differentiate toward both the chondrogenic and the osteogenic lineage similarly to the bone marrow MSCs [40, 41]. Indeed, like the bone marrow counterpart, UC-MSCs react to low oxygen tension conditions [42] or to pulsed electromagnetic fields [43] showing an improved chondrogenic commitment. The promising multidifferentiation potential may be due to the peculiar site of origin leading to a “more embryonic” feature than the bone marrow counterpart [14, 44, 45]. Indeed, they have been recently used *in vivo* as described by Sadlik et al. [46] involving the dry arthroscopic treatment of cartilage lesions with UC-MSCs embedded in a porcine type I/II collagen matrix. Dilogo et al. [47] also used UC-MSCs for the treatment of a critical-sized bone defect; however, literature suggests caution before a widespread clinical application, claiming for a better understanding of the functional characteristics of these cells [10]. Nonetheless, a recent evidence has added a further promising opportunity in the field of UC-MSCs. In recent studies, Mennan et al. [48] and Hendijani et al. [49] have demonstrated that a mixed cell population obtained by processing the whole umbilical cord seems to have the same differentiation potential than cells derived from single areas of the cord (Wharton's jelly, artery, vein, or cord lining). These findings might suggest the possibility to obtain optimal precursor cells without any concern about the separation of specific population, simplifying any theoretical use of the cord as a source of cells for future hypothetical widespread clinical application.

In line with these perspectives, our study has analyzed the *in vitro* chondrogenic and osteogenic commitment of a mixed UC-MSC population, obtained with a very simple and economic protocol without any enzymatic digestion. No cell selection has been performed during the MSC extraction from cord stroma, but the adherent properties of UC-MSCs and the simple mincing of the cord fragments represented the essential steps of our cell isolation method. This choice is in line with the aforementioned evidences in literature [48, 49], and it allows for greatly simplifying the cell harvesting without reducing the efficiency of the protocol, as recently outlined by Yoon et al. [50].

Both human platelet lysate obtained from healthy donors and fetal bovine serum (FBS) were used for culture, as described in a previously published paper [15], in order to optimize the broth conditions for the cells. For a human hypothetical application, the FBS may arise ethical concerns [51]. Thus, the choice of using human platelet lysate only seems to be preferable for future human trials. Further study might compare the efficacy of this method with and without FBS to ensure that an adequate number of cells may be obtained in both conditions.

Indeed, our protocol led to a consistent number of cells per gram of tissue, thus suggesting a possible future large-scale nonexpensive cell storage for clinical purposes, as Kalaszczynska and Ferdyn envisioned in a recent review [8].

UC-MSCs obtained with our method have shown stemness properties in terms of markers and telomere preservation, the latter suggesting a maintenance of cell viability, as recently outlined in literature [8]. Moreover, UC-MSCs showed a low expression of HLA-I and no expression of HLA-II, consistent with a possible safe allogeneic use. This concept is confirmed by a recent study by Liu et al. [44] in which the authors demonstrated that the immunoprivileged status of UC-MSCs seems to be maintained even during the differentiation process toward a specific mesenchymal (i.e., chondrogenic) lineage. Furthermore, the finding of an “HLA-I and HLA-II double negative” cell subpopulation suggests peculiar immunoprivileged properties of these cells that may deserve future studies to verify the possible isolation of this cellular subtype, their specific differentiation potentials, and their hypothetical elective use as preferential candidate for allogeneic therapies.

To test UC-MSC chondrogenic commitment in a tridimensional environment, two widely used scaffolds have been compared (Chondro-gide and Hyaff). We have observed cell growth on both membranes, with differences in cell distribution depending on the tridimensional scaffold structures. Nevertheless, in both experimental groups, cells showed a chondrogenic phenotype and they stained positive for the chondrogenic markers sox-9 and collagen type II. Notably, the low oxygen tension exerted an influence on UC-MSCs, similar to bone marrow-derived MSCs [52]. Specifically, low oxygen tension did not hamper UC-MSC proliferation potential, but it led to an increased matrix production in UC-MSC pellet cultures and to an enhanced chondrogenic marker expression in UC-MSC scaffold cultures, consistent with previous studies [42]. This increased chondrogenic differentiation might be the result of hypoxia-inducible factor-1 alpha and factor-2 alpha stabilization and the subsequent sox-9 induction [53–56]. Further in vitro analyses are necessary to better clarify this specific aspect. Overall, a clear chondrogenic commitment has been observed with both scaffolds, with a slight greater evidence of collagen II production when UC-MSCs were grown on a hyaluronic acid membrane. This is consistent with several evidences in literature that showed a possible and efficient chondrogenic differentiation of mesenchymal stem cells derived from Wharton's jelly in different conditions, as the high-density cell cultures on rotatory systems [57], the culture in collagen hydrogels [58], or the cell growth on polycaprolactone/collagen nanoscaffolds [40].

Like the chondrogenic differentiation, the osteogenic commitment was obtained using a commercial organic bone substitute used for clinical applications, to closely mimic the clinical application. Indeed, the common bovine-derived bone matrix used in this study is widely accepted in clinical scenarios as gap filling in the presence of bone defects. We have observed an osteogenic potential similar to other studies where cells from umbilical cord were grown in monolayer [15] or in tridimensional scaffold as collagen I/III gels [59] or even when loaded onto bone scaffold and subcutaneously implanted in nude mice [60]. Our results, along with the available literature, suggest that UC-MSCs might represent a possible source allogeneic cells for bone tissue engineering.

These observations are further confirmed by the last section of our experiments. The idea of these experiments has been inspired by Scotti et al. [61], who conceived a complex preclinical model to demonstrate the feasibility of engineering a functional bone organ by preconditioning bone marrow-derived MSCs, embedded in type I collagen meshes, toward hypertrophic chondrocytic phenotype and subsequently implanting the scaffolds in the dorsal subcutaneous tissue of nude mice. The dramatic originality of Scotti's study and the potential for bone engineering are unquestionable. Indeed, bone repair mainly occurs through endochondral ossification and the possibility to recapitulate this process may allow for conceiving a “second generation” cell-seeded bone substitutes with increased efficiency to strongly facilitate bone repair and regeneration. In our study, we verify *in vitro* the UC-MSC ability to execute an endochondral program similar to BM-MSCs and we applied the same conditions previously described by Scotti et al. [61]. We seeded UC-MSCs onto the previously mentioned commercial bone substitutes, and we cultured the cell-seeded constructs in chondrogenic conditions and subsequently in the hypertrophic medium. We obtained encouraging results in terms of matrix synthesis and expression of osteogenic markers (Cbfa1). This certainly represents one of the most original features of our study. Moreover, our work represents one of the first studies describing the use of UC-MSCs in a “developmental engineering” paradigm. Indeed, the positive results observed by “pushing” UC-MSCs through endochondral ossification may be exploited by future experiments verifying the attitude of these cells to generate a tissue similar to cancellous bone after *in vivo* preclinical implantation in small animals, in order to offer a valid use of allogeneic cell-seeded bone substitutes.

The main limitation of this study is the *in vitro* nature of the experiment. This certainly claims for further studies to verify the intuitions derived from the obtained data in preclinical animal models before moving to any clinical applications. Albeit this drawback is implied in any *in vitro* study, the preclinical model is essential when dealing with allogeneic cells to assess the immune privilege of this category of stem cells before any *in vivo* human use. However, literature offers several evidences of UC-MSC immunoprivileged status. These cells express HLA-G, which is involved in immune tolerance during pregnancy IL-6 and VEGF, which are linked to MSC immunosuppressive capability. Moreover, UC-MSCs are able to suppress T-cell proliferation [14]. All these aspects seem to confirm the potential for an allogeneic use of UC-MSC in orthopaedic tissue engineering.

Nevertheless, a critical issue when dealing with allogeneic MSCs remains the potential for immune reaction to be elicited in the host tissue. Evidences in literature are present outlining the immunogenicity of MSCs from an allogeneic source and the fact that this reaction may hamper dramatically the tissue regeneration process in the same cells were supposed to be enhanced. The study of Eliopoulos et al. in 2005 [62] showed the alarming reject of allogeneic MSCs implanted subcutaneously in mice. A similar fate of these cells has been observed by Huang et al. [63] in a preclinical rat model of myocardial infarction. In their study, the authors showed that allogeneic cells were rejected from the host cardiac tissues by 5 weeks after implantation. This may be partially explained by a transient immunoprivileged status of MSCs that may later acquire immunogenicity during their differentiation and trophic process in the host environment, thus eliciting an immune reaction by the native immune system. Immune reactions and short engraftment time have also been described by Tano et al. [64] in a model of allogeneic MSC transplantation on the epicardial surface of rat hearts. A similar paradigm has been confirmed recently by Oliveira et al. [65] in a preclinical model of MSC transplantation in the murine kidney. They observed lymphocytic infiltration and allogeneic MSC rejection no later than 28 days after transplantation. As a consequence, they suggested the fascinating concept of “preactivation” of cells, by treatment with INF-gamma and TNF-alpha, in order to reduce immunogenicity and prolong their engraftment period and their trophic properties. A potential immune reaction in human has been recently hypothesized by de Windt et al. [31] in their clinical trial of cartilage repair by means of allogeneic MSCs and chondrons. Despite their encouraging results, no allogeneic cells were found at the repair site at 1 year, suggesting a time-limited trophic effect of MSCs that would have been gradually removed by the host immune system during their differentiation processes. This agrees with a previous in vitro observations of Mukonoweshuro et al. [66], who have outlined the immunosuppressive properties but not the immunoprivileged status of allogeneic chondrogenic-committed MSCs in a mouse model. The potential immune reaction elicited by MSCs may also lead to detrimental effect in the repair setting, as shown preclinically by Sbano et al. [67] in a mouse model of skin graft transplantation and by Seifert et al. [68] in a rat kidney transplantation setting. Overall, these evidences claim for a cautious approach in a potential clinical application of allogeneic MSCs, despite the recent literature is outlining, as a counterpart, the safety and some promising results. Indeed, the studies of Garcia-Sancho et al. [69] showed a possible application in knee osteoarthritis and degenerative disc disease with few safety concerns, probably due to the peculiar “insulated” recipient site of the cells, and Wang et al. [70] have recently proposed the injection of allogeneic MSCs during ACl reconstruction to improve symptoms and structural outcome. With a longer follow-up, Park et al. [71] have shown the promising effect of the implantation of a composite made by allogeneic UC blood-derived MSCs and hyaluronate hydrogel to improve cartilage healing in osteoarthritic patients. Similar evidences have been proposed by Vega et al. [72] and by Gupta et al. [73], who treated knee osteoarthritic patients by a direct intra-articular injection of allogeneic human MSCs. In a different setting, Morrison et al. [74] have recently demonstrated the feasibility of cranial bone reconstruction by means of allogeneic mesenchymal stromal cells (MSCs) on a ceramic carrier and polymer scaffold, similarly to the preclinical observations of Todeschi et al. [75] regarding UC-MSC transplantation in mice calvarial defects. So far, the controversy of allogeneic MSC therapy is still a debated issue in literature. However, a common element seems to be present in all these studies. Indeed, a different role of MSCs is emerging from the preclinical and clinical recent evidences that may lead to reconsider the use of allogeneic MSCs as “replenishing cells for injured tissues,” assuming their immune system recognition. Far from being a “building block” submitted to the host immune system (and, thus, with a limited viability of 2–4 weeks), the allogeneic MSCs may otherwise represent the transient paracrine catalyzers that work well for a short period of time with a “hit and run effect” [65]. This new paradigm implies the lack of the “need of persistence” in the host tissue, as the therapeutic effects of MSCs are independent of a direct differentiation, but they can be exerted locally by promoting the resident population to hasten the repair process for a limited period of time, until the allogeneic MSCs are cleared by the host immune system. In this perspective, it can be easily understood the greater effect of implanting allogeneic MSCs in a “confined environment” (i.e., in the joint or embedded in a bioscaffold); as in these settings, the immune system may be hampered in rapidly eliminating the MSCs. Furthermore, the various regenerative properties of allogeneic MSCs observed in literature may coexist with a reduced survival time due to the fact that they depend on the factors secreted by the cells in the first days after transplantation, also known as the “secretome.” Indeed, together with the concept of MSC preactivation to prolong the cell survival time, the secretome surely represents one of the future frontiers in allogeneic MSC tissue engineering research.

In conclusion, the present study confirms the chondrogenic and osteogenic commitment of UC-MSCs when cultured in tridimensional scaffold and it suggests the possible involvement of UC-MSCs for the generation of endochondral scaffolds to improve bone regeneration by recapitulating endochondral ossification process. These observations offer a solid perspective for future preclinical studies aiming to improve cartilage repair and bone regeneration. Furthermore, UC-MSCs derive from a previously discarded material as the umbilical cord; thus, they represent a stem cell population with several advantages as a greater bioavailability and lower ethical implications than other cell sources. All in all, UC-MSCs may be reasonably considered an attractive opportunity for orthopaedic allogeneic stem cell therapy.

## Supplementary Material

Supplemental Figure 1: Cell count and expansion at P1. Supplemental Figure 2: Flow cytometry analysis of surface-marker expression on umbilical cord mesenchymal cells, after one passage in culture. Supplemental Figure 3: Chondrogenic differentiation of UC-MSCs seeded on Chondro-gide. Supplemental Figure 4: Chondrogenic differentiation of UC-MSCs seeded on Hyaff. Supplemental Figure 5 Expression of osteogenic markers in UC-MSCs seeded onto Orthoss cubes.
