# Supplementary material for: Allogeneic Umbilical Cord-Derived Mesenchymal Stem Cells as a Potential Source for Cartilage and Bone Regeneration: An In Vitro Study
Source: Stem Cells Int. 2017 Nov 16;2017:1732094. doi: 10.1155/2017/1732094 (PMC5735324; doi:10.1155/2017/1732094)
Supplement: Supplementary file 1 — Supplemental Figure 1: Cell count and expansion at P1. Supplemental Figure 2: Flow cytometry analysis of surface-marker expression on umbilical cord mesenchymal cells, after one passage in culture. Supplemental Figure 3: Chondrogenic differentiation of UC-MSCs seeded on Chondro-gide. Supplemental Figure 4: Chondrogenic differentiation of UC-MSCs seeded on Hyaff. Supplemental Figure 5 Expression of osteogenic markers in UC-MSCs seeded onto Orthoss cubes. [file 1732094.f1.pdf]

Sample #	Age of donor	Sex of the child	Umbilical cord weight (gr)	Umbilical cord length (cm)	n° cells at P1 x 10^6	cells x 10^6 at P1/gr tissue
1	29	M	26.30	23.50	21.60	0.82
2	39	F	23.90	38.50	0.00	0.00
3	38	M	44.50	75.00	24.00	0.54
4	28	F	39.80	58.00	25.00	0.63
5	36	F	16.50	20.00	23.00	1.39
6	30	M	66.00	51.00	24.00	0.36
7	24	M	32.30	34.50	21.00	0.65
8	34	M	22.10	46.00	25.00	1.13
9	41	F	20.20	35.00	0.00	0.00
10	33	M	25.10	37.00	0.00	0.00
11	25	M	30.10	45.00	0.00	0.00
12	32	F	32.30	32.00	22.00	0.68
13	34	M	26.20	37.00	23.00	0.88
14	30	M	23.20	45.00	21.00	0.91
15	39	F	31.20	36.00	24.00	0.77
**Standard Dev.**	5.19		12.20	13.55	10.63	0.44
**MEDIA**	32.80		30.65	40.90	16.91	0.58
