# Supplementary figures and images for: Allogeneic Umbilical Cord-Derived Mesenchymal Stem Cells as a Potential Source for Cartilage and Bone Regeneration: An In Vitro Study
Source: Stem Cells Int. 2017 Nov 16;2017:1732094. doi: 10.1155/2017/1732094 (PMC5735324; doi:10.1155/2017/1732094)

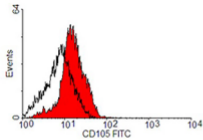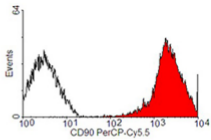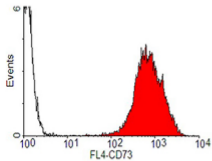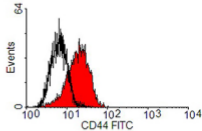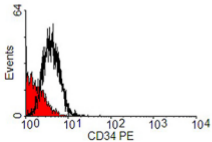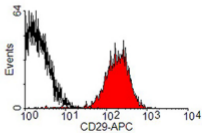

Supplement: Supplementary file 2 [file 1732094.f2.pdf]

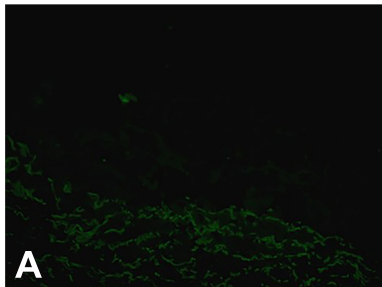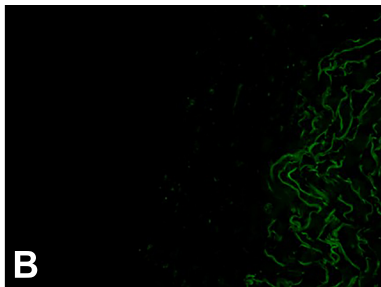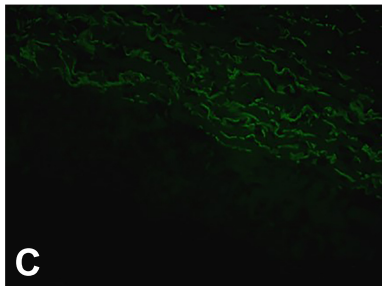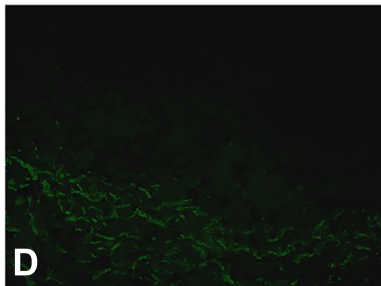

Supplement: Supplementary file 3 [file 1732094.f3.pdf]

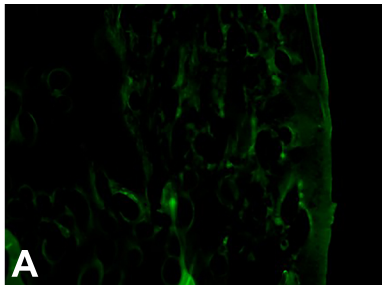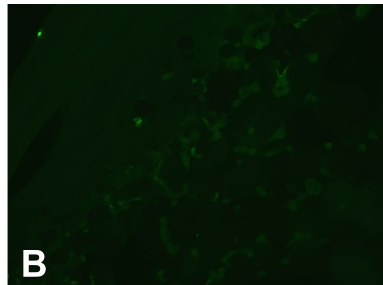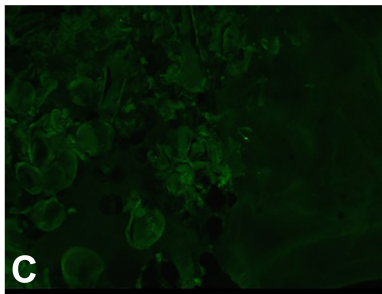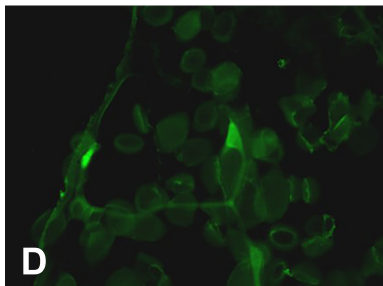

Supplement: Supplementary file 4 [file 1732094.f4.pdf]

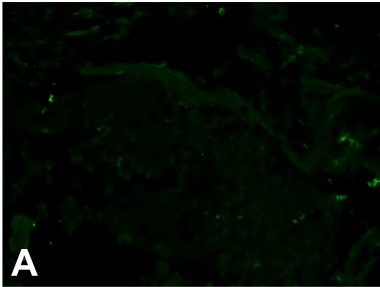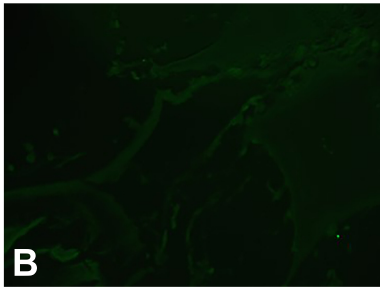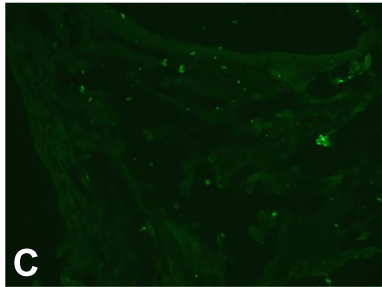

Supplement: Supplementary file 5 [file 1732094.f5.pdf]
